# Lessons from inter-disciplinary collaboration to mitigate SARS-CoV-2 transmission in schools, Ireland, 2020/2021, to inform health systems and multisectoral recovery

**DOI:** 10.3389/fpubh.2022.1072566

**Published:** 2023-01-16

**Authors:** Peter Naughton, Ciara Kelly, Philippa White, Elizabeth Kennedy, Anne Healy, Abigail Collins, Mary Ward

**Affiliations:** ^1^Department of Public Health, Area B, Dr. Steevens' Hospital, Dublin, Ireland; ^2^PricewaterhouseCoopers, Dublin, Ireland; ^3^Quality of Care Unit, Department of Integrated Health Services, World Health Organization, Geneva, Switzerland; ^4^Department of Public Health, Area C, Dr. Steevens' Hospital, Dublin, Ireland; ^5^Child Health Public Health Programme, Health Service Executive, Tullamore, Ireland

**Keywords:** COVID-19, Ireland, schools, multisectoral, health systems, recovery, resilience, interdisciplinary

## Abstract

**Introduction:**

School closures associated with the COVID-19 pandemic resulted in the loss of educational and social supports for up to 1,000,000 students in Ireland and disproportionately affected students from lower socio-economic backgrounds. For the 2020/2021 school year, multisectoral and interdisciplinary “Schools Teams” were established within Public Health departments to maintain in-person education by minimizing transmission of SARS-CoV-2 in schools. This study aimed to describe this model and explore the experiences of Schools Team members in the East of Ireland to identify factors that influenced effective working that can be sustained in the context of health systems and multisectoral recovery.

**Methods:**

Schools Teams were comprised of multidisciplinary staff from regional Public Health departments and redeployed staff from the Education sector. Governance rested with Public Health departments. All staff operated to nationally agreed protocols following training. The experiences of the East Schools Team members were explored through an online survey and semi-structured interviews.

**Results:**

The survey response rate was 53/70 (75.7%). Participants reported clear channels of communication within the team (44, 83.0%), feeling comfortable in their role following training (43, 82.7%) and a positive team culture (51, 96.2%) as key facilitators of effective inter-disciplinary working. Insufficient administrative support and mixed messaging to schools were identified as barriers to efficient team collaboration.

**Discussion:**

The Schools Team model illustrates the potential for multisectoral partnerships to effectively address complex public health priorities and contribute toward health system resilience to health threats. By recognizing and leveraging the ability of allied sectors such as the education sector, to contribute to public health goals, countries can move toward the kind of whole-of-government approach to health recognized as key to health system resilience. The strong links between the education and public health sectors developed through this collaboration could be extended and strengthened to more effectively pursue public health priorities in school settings. More broadly, mechanisms to support multisectoral working should be developed, expanding beyond reactive interventions to proactively address key health priorities and build resilience across health systems and communities. Such collaborations would promote healthier populations by promoting and encouraging a public health perspective among other sectors and embedding “health in all policies”.

## 1. Introduction

Coronavirus disease 2019 (COVID-19), the disease caused by the severe acute respiratory syndrome coronavirus 2 (SARS-CoV-2), was declared a pandemic by the World Health Organization (WHO) on 11 March 2020 (1).^1^ Since then, countries worldwide have introduced a range of public health measures throughout the pandemic to control transmission of SARS-CoV-2.

School closures are one such measure, which have occurred in over 200 countries and territories globally to date, impacting millions of students (2).^2^ These closures were often enacted in the early phase of the pandemic when the role of schools in SARS-CoV-2 transmission was uncertain (3). However, multiple studies have since demonstrated that schools are not drivers of SARS-CoV-2 transmission (4, 5). Instead, the incidence of COVID-19 in schools has largely followed that of the local community (6–9).

School closures drastically and rapidly altered the learning context for children worldwide since their introduction in 2020 resulting in the loss of educational and psycho-social supports (10). Despite many settings switching to online learning, school closures have deepened inequalities in education, with a disproportionate impact on children from lower socio-economic backgrounds who are less likely to have access to the prerequisites of effective online learning, e.g., computers, internet access and quiet learning environments. Leading health organizations have recommended that school closures should be used as a last resort to control COVID-19 transmission due to the adverse effects of these measures on children's physical and mental health (11, 12).^3^

In Ireland, pandemic related school closures were first introduced on 12 March 2020 as part of a range of public health restrictions. Schools did not re-open for the remainder of that school year to June 2020 (13).^4^ By September 2020, increasing COVID-19 case numbers prompted the re-introduction of many public health control measures, including the closure of many retail shops, restaurants, bars, gyms and limiting public transport to 25% capacity (14).^4^ However, unlike during the previous wave of infection, schools were not closed.

To support schools to remain open and develop a more resilient response to COVID-19 in educational settings (i.e., primary, secondary and special schools), dedicated multisectoral and interdisciplinary “Schools Teams” were established within each of the eight regional Public Health departments.

The COVID-19 pandemic has caused widespread disruption to health systems and placed increased demands on finite health system resources. However, there is a paucity of literature describing the potential of multisectoral and interdisciplinary collaboration to successfully address complex public health priorities. Therefore, there is a need to increase the evidence base for such interventions and promote awareness of this model among public health practitioners, health system managers and policy makers.

The aims of this study were as follows:

1. To explore the experiences of Schools Teams members in the East of Ireland to identify factors that influenced effective inter-disciplinary working.
2. To discuss how the lessons learnt from the Schools Team model can inform future multisectoral collaborations to address complex public health priorities.

## 2. Methods

### 2.1. Schools team structure

Schools Teams were comprised of staff from departments of Public Health and staff redeployed primarily from the government Departments of Education and of Children, Equality, Disability, Integration and Youth. The Schools Team framework was developed at a national level and supported at government ministerial level. Clinical and data governance structures were agreed between stakeholders. Regular communication occurred between national Public Health and Education sector leadership to ensure understanding and confidence in the agreed processes between all stakeholders.

Schools Teams operated according to nationally agreed protocols to respond to cases and outbreaks of COVID-19 in educational settings. Once a COVID-19 case was identified in a student or school staff member, the case was referred to the Schools Team, who contacted the educational setting and performed a public health risk assessment. The objectives of the risk assessment included:

- To determine if a case attended school while infectious, i.e., within 48 h of symptom onset or 24 h of the test date if asymptomatic.
- To consider whether a case was likely infected in the community or part of a school outbreak.
- To determine the close contacts of the case, provide them with appropriate public health advice and refer them for testing *via* a dedicated pathway.
- To support schools in the practical implementation of infection prevention and control guidelines.

### 2.2. Study design

A mixed methods investigation was designed to identify the specific barriers and facilitators to effective team working among Schools Team members in the East region of Ireland. This team was chosen as it was the largest individual Schools Team (70 members) and covered 32% of the Irish school aged population. The experiences of team members were explored through an online questionnaire. Subsequently, interviews with randomly selected individuals were conducted to gain a deeper understanding of specific topics identified from questionnaire responses.

#### 2.2.1. Questionnaire design

Draft questions were formulated in consultation with a core group of experienced Schools Team members and three pilot interviews were conducted to assess the questionnaire for content and face validity.

The questionnaire contained both open and closed-ended questions. Closed-ended questions were assessed by asking respondents if they agreed with a given statement using a five-point Likert scale. A “not applicable” (NA) option was also included as a potential response for each categorical question. A copy of the questionnaire is available in Supplementary material 1.

All Public Health and Education Schools Team members were contacted by e-mail and provided with information about the study and a link to complete the questionnaire. Online questionnaires were administered using the Jotform survey platform (15).^5^ All survey data were collected between 22 December 2021 and 10 February 2022.

#### 2.2.2. Interview design

Complex topics which required more in-depth understanding were identified from questionnaire responses and explored through individual semi-structured interviews. An interview guide was developed to ensure that core themes were covered in all interviews while still allowing flexibility to explore particular issues in line with participants' experiences.

Four team members (two each randomly selected from both the Public Health and Education sectors) were invited to participate in the interview process. All interviews were conducted over video call by a lead public health nurse with extensive experience in communications. The average interview duration was 42 min (range 30–53 min). Interviews were recorded and transcribed using the auto-transcribe feature of Microsoft Teams for Windows (version 1.5.00.9163). Each transcript was manually reviewed by the lead author for accuracy.

### 2.3. Study population

All staff who worked on the East Schools Team during the 2020/2021 academic year were invited to participate in the online questionnaire, regardless of their duration of service.

### 2.4. Data analysis

Descriptive statistics were generated using R statistical software and data visualization was carried out using the package ggplot2 (16, 17). Thematic analysis was performed on open-ended responses (18). These responses were reviewed to identify the individual topics raised by each participant. Initial codes were generated for each topic and similar codes were grouped together to identify emerging themes relevant to the study question. This process was repeated several times as more responses became available. The data were examined by the lead author to identify patterns in the distribution of themes among participants and potential relationships between different themes. Data were analyzed using Nvivo – Mac (version 1.6.2) (19).^6^

### 2.5. Ethics

Ethical approval was not required as this work was a retrospective service evaluation of a public health response conducted under Infectious Diseases Regulations (1981) (20).^7^ All collected data was anonymous and work conducted in line with ethical and data protection principles.

## 3. Results

### 3.1. Survey respondent characteristics

All 70 East region Schools Team members were invited to complete the online questionnaire. In total, 53 questionnaires were returned, resulting in a response rate of 75.7%.

**Figure 1:**
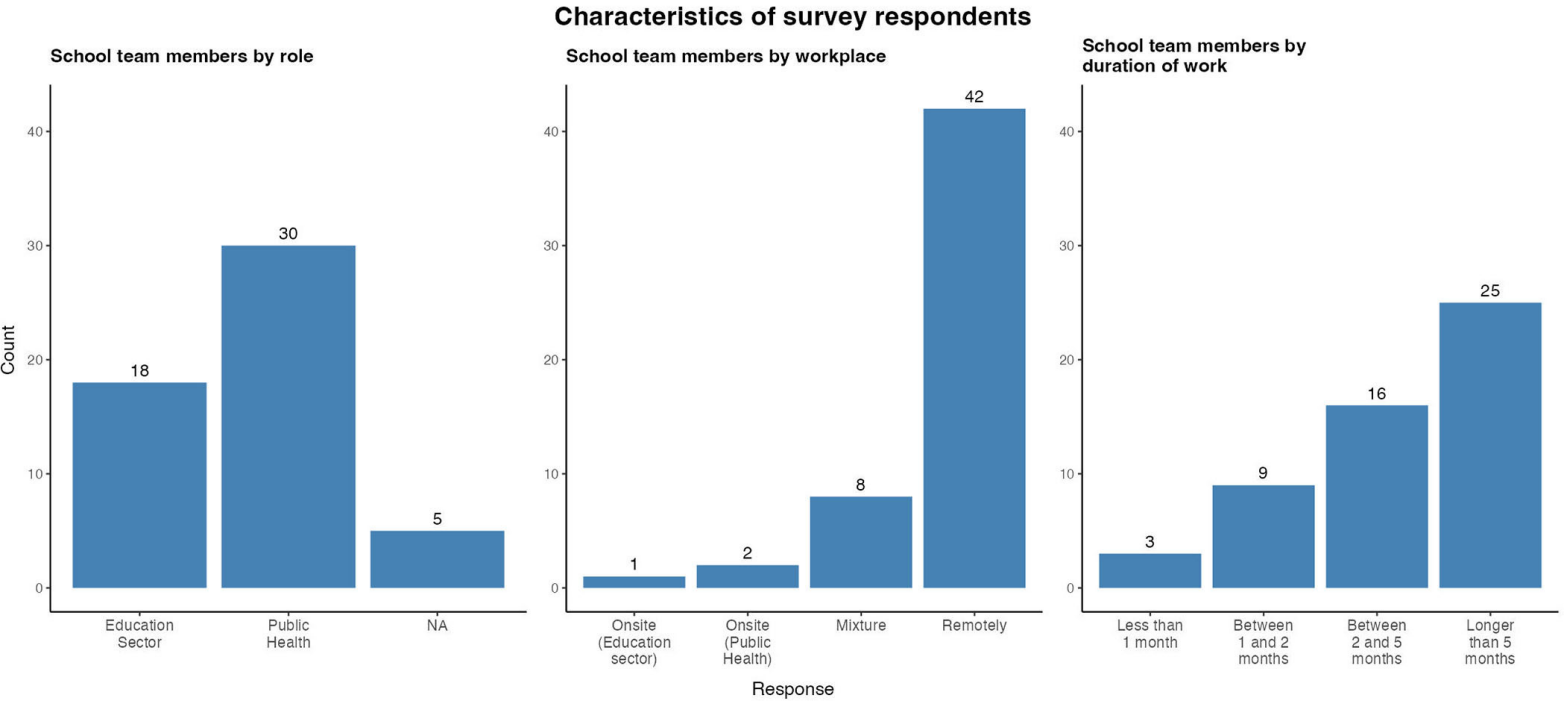
Respondent characteristics of East Schools Team survey, Ireland, 2020/2021 academic year.

Public Health staff comprised a higher proportion of respondents (30, 56.7%) compared to Education sector staff (18, 34.0%). No affiliation was declared by 5 (9.4%) participants (Figure 1). The majority of respondents (42, 79.2%) worked completely remotely and almost half (25, 47.2%) of the respondents had worked with the Schools Team for longer than 5 months.

### 3.2. Data analysis

Following analysis, data from open and closed ended survey questions and semi-structured interviews were grouped into three broad themes: communication, team organization and team culture. Findings concerning each of the broad themes are presented below.

### 3.3. Communication

**Figure 2:**
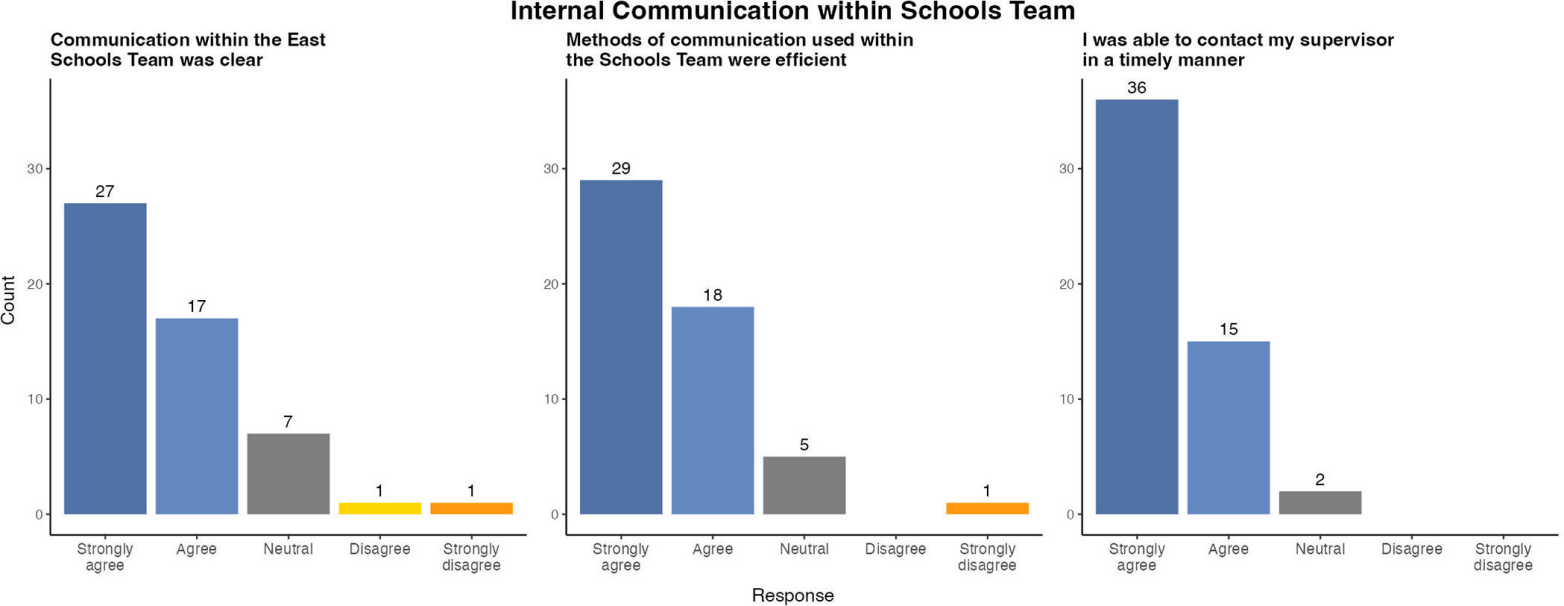
Survey results of evaluation of internal communication within East Schools Team, Ireland, 2020/2021 academic year.

Clarity of communication emerged from questionnaire responses as one of the key facilitators of effective team working (Figure 2). The majority of team members (44, 83.0%) agreed or strongly agreed that communications within the Schools Team were clear. Team members reported that the methods of communication used (phone calls, regular e-mail updates, daily virtual team meetings) efficiently disseminated information throughout the team. Several respondents also stated that daily meetings were a source of “moral support” as well as clinical guidance. The switch from telephone conference calls to video calls was identified as improving the clarity of communication between colleagues when working remotely. Almost all (51, 96.2%) team members agreed or strongly agreed that they had timely access to senior support as required.

“*The effective communication across multidisciplinary teams, for me, was the most impressive part of working on the team.”*

“*Excellent system for keeping in regular contact with the team, despite a lot of the work occurring remotely.”*

**Figure 3:**
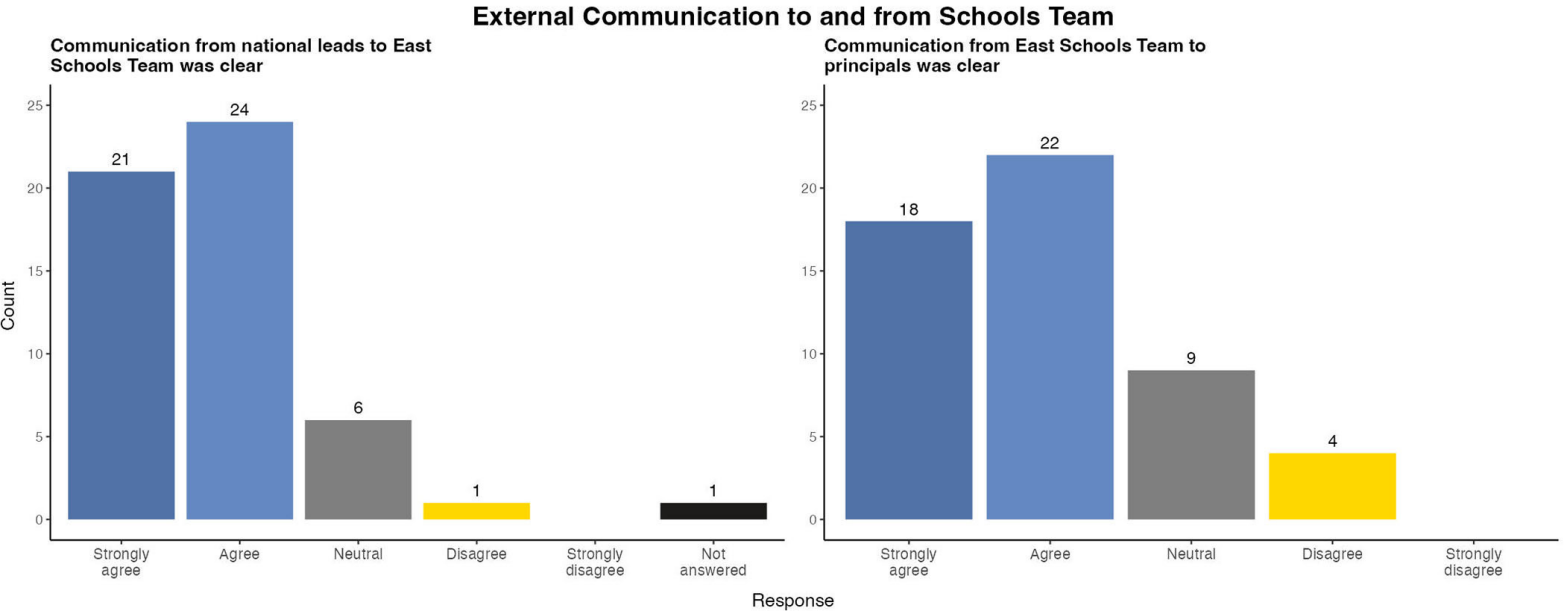
Survey results of evaluation of external communication to and from East Schools Team, Ireland, 2020/2021 academic year.

Respondents were broadly satisfied that updates from the national schools leads were clearly disseminated to the East Schools Team (Figure 3). Many respondents credited the multisectoral composition of the team with improving the effectiveness of communication with school principals. Several Education sector staff reported that their knowledge of the school environment and existing rapport with principals allowed them to provide more relevant guidance.

“*The communication was understood and translated by the Department of Education staff in a way to principals that made sense.”*

However, 9 (16.9%) participants felt neutral and 4 (7.5%) disagreed with the statement that messaging from the Schools team to principals was clear. This finding was explored in individual interviews which identified media reports and rumors of potential changes in COVID-19 guidance as barriers to communication with principals.

“*Communications [with principals] were often confusing as there appeared to be conflicting advice coming from different quarters. A lot of time was spent clarifying queries which emanated from disinformation that principals had encountered in various quarters.”*

Delays in receiving positive case notifications or duplicate notifications were also highlighted as barriers to effective communication with principals. Several respondents attributed these issues to insufficient administration staff.

“*Unfortunately, sometimes there was mass duplication in the system and principals became frustrated when they received multiple calls from the Schools Team.”*

### 3.4. Team organization

**Figure 4:**
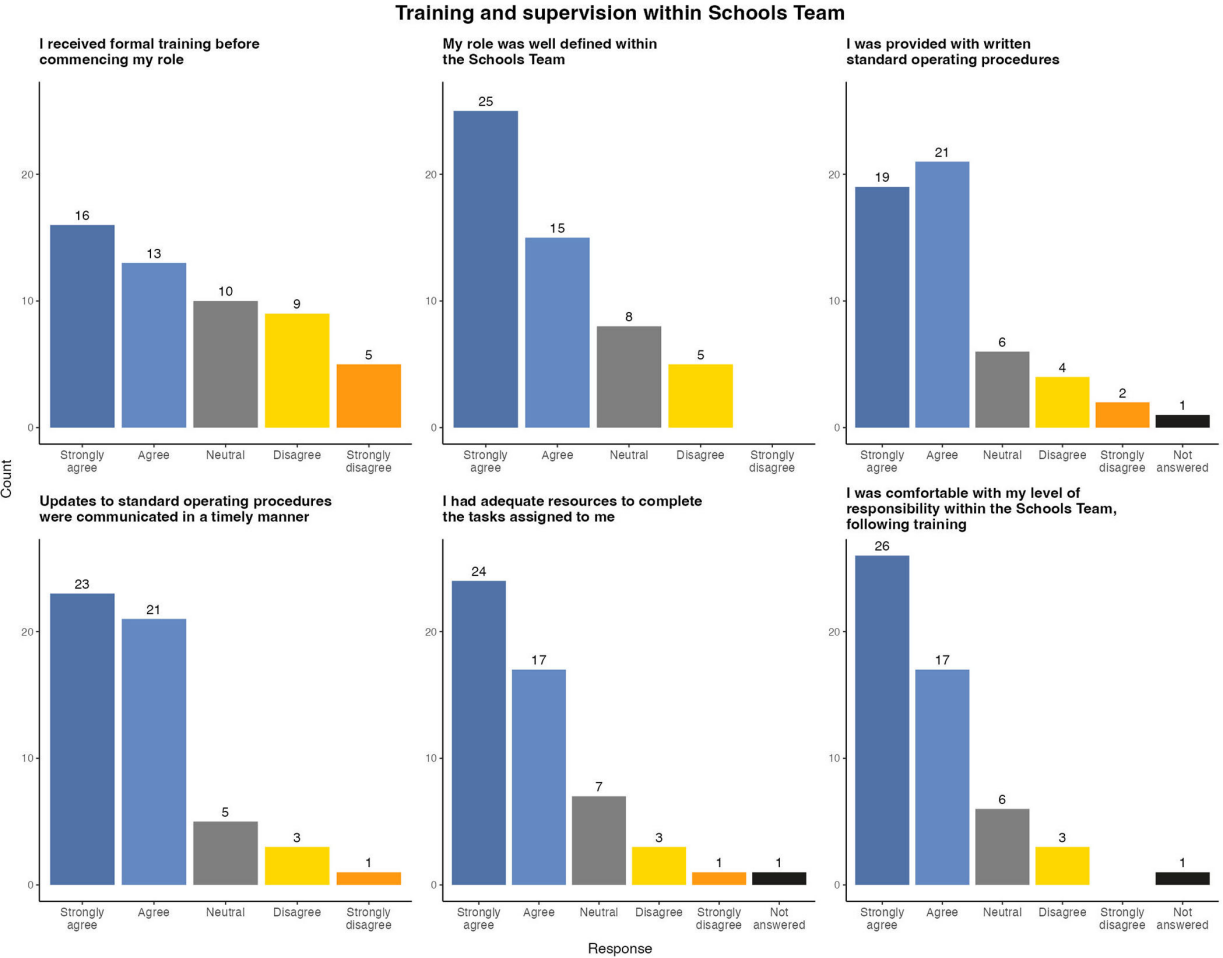
Survey results of evaluation of team protocols and training within East Schools Team, Ireland, 2020/2021 academic year.

Clear and concise standard operating protocols were regularly identified by participants as facilitating effective team working. Team members reported that written protocols helped to define their roles and responsibilities and provided clarity on who to contact if they required support. Respondents stated that standardized protocols ensured a consistent approach across all settings and helped to empower them when communicating advice to principals. However, some respondents stated that the high frequency of protocol updates was challenging to implement, especially when principals had become familiar with a previous protocol iteration. The majority agreed that all updates were disseminated to the Schools Team in a timely manner (Figure 4). These findings were echoed in open ended questionnaire responses.

“*Well written protocols, clear from an operational perspective.”*

“*The visual algorithms were extremely useful for new recruits to the Schools Team.”*

“*Updated iterations communicated to all team members in a timely manner; demonstrations undertaken when there were any major updates.”*

Team members reported that standardized protocols facilitated efficient working and increased the number of case notifications which they could process per day. Despite these efficiencies, some respondents found managing the high expectations of schools to be challenging, for example receiving phone calls from schools outside of normal working hours.

Formal training in standard operating protocols, team organization and methods of communication within the Schools Team was identified as a concern by some respondents. In total, 14 (26.4%) respondents disagreed or strongly disagreed that they received adequate training prior to commencing work with the East Schools Team. Many of these respondents joined the team later in the academic year and reported receiving helpful “on the job” training instead which involved shadowing existing team members. While new team members found this training useful, it led to an increased work load for the experienced team members conducting training.

“*The opportunity to shadow colleagues was very helpful and important.”*

“*I think we got a lot of training at the very beginning, but once new team members arrived, they got a lot less and we had to take on their training.”*

However, following the completion of training the majority of team members felt comfortable with their role and responsibilities in the Schools Team.

### 3.5. Team culture

**Figure 5:**
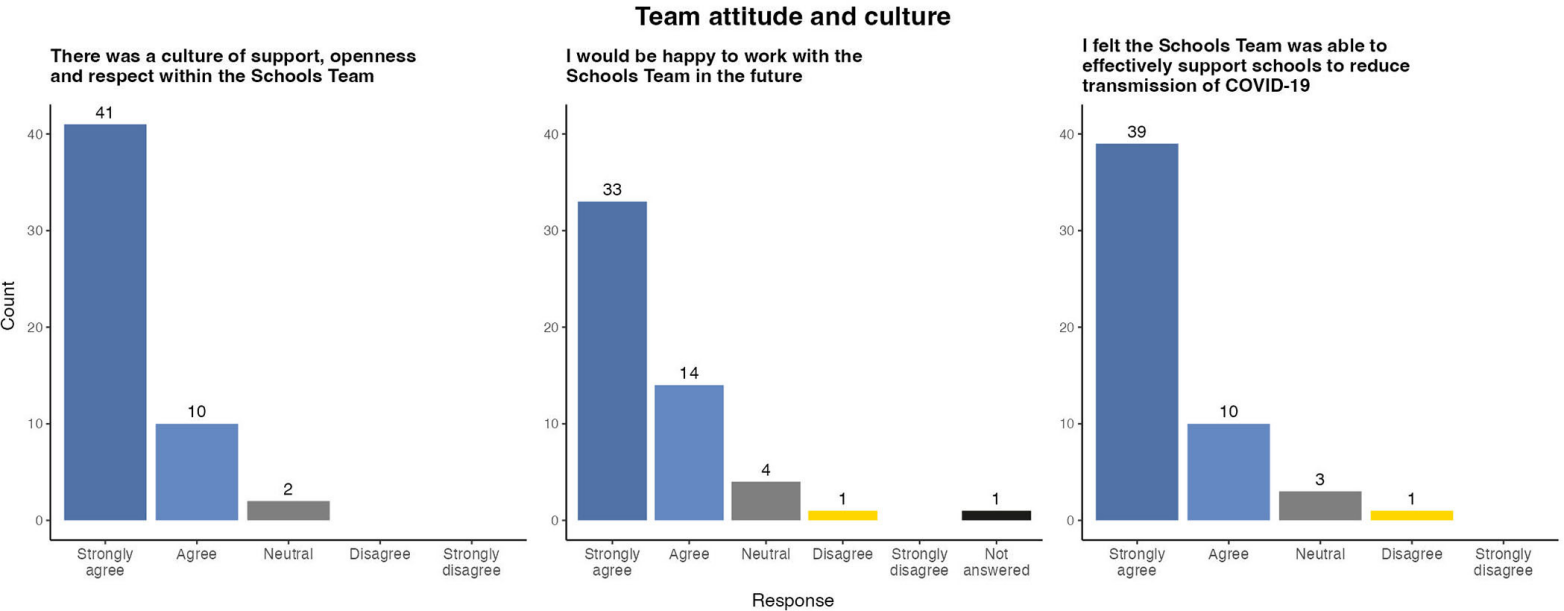
Survey results of evaluation of team culture within East Schools Team, Ireland, 2020/2021 academic year.

A positive team culture was highlighted by both Public Health and Education sector staff as one of the strongest facilitators of effective working (Figure 5). The majority (41, 77.4%) of respondents strongly agreed that there was a culture of support, openness and respect within the Schools Team and this was reflected in open ended responses.

“*It was a lovely culture, was lovely and supportive, you know, everybody got along. It was a great team vibe.”*

“*It was a very, very enjoyable working environment.”*

Despite working largely remotely, a number of respondents referenced the positive working relationships which developed between staff from various disciplines over the academic year.

“*I suppose the thing that stood out for me really was the relationship that built up between the Department of Education people and Public Health.”*

“*For me, the thing that worked the best was the really positive good collaborative relationships.”*

Team members stated that the collaborative team atmosphere was especially helpful when dealing with complex cases.

“*I felt we weren't doing this in isolation or you weren't just stuck in your room or your office, but that there was a community there around you and supporting you.”*

When asked about the effectiveness of the Schools Team, 39 (73.6%) respondents strongly agreed that they felt they had successfully mitigated SARS-CoV-2 transmission in schools. Several respondents linked this sense of achievement as contributing to a positive team environment.

“*There was a sense that we were contributing to the 'cause' and I think we all saw that as something very positive.”*

As a result of their positive experience, the vast majority of respondents stated that they would be happy to work with the East Schools Team again in the future.

## 4. Discussion

### 4.1. Consideration of key findings

These findings provide a clear insight into the human and organizational factors which promoted effective multisectoral and interdisciplinary work within the East Schools Team.

Open channels of communication were a key facilitator of early team integration, despite the majority of team members working remotely. Regular team meetings allowed the majority of issues to be addressed proactively, and accessible senior support allowed for timely discussion of urgent issues. Standardized protocols improved workflow efficiency by enabling team members to work with as much autonomy as possible, while accessing senior support as needed. When combined with training, this increased autonomy empowered all individuals (clinical and non-clinical) to maximize their contribution to the Schools Team and reduced barriers between staff from different sectors.

The multisectoral model enhanced the effectiveness of the Schools Team by leveraging the strengths of each sector. Public Health staff ensured that team protocols reflected the national guidance and optimal health protection approach to SARS-CoV-2 mitigation while Education sector staff utilized their knowledge of the practical challenges faced by principals to effectively implement guidance at local school level. A positive team culture also played a key role in fostering a proactive and unified team atmosphere. Team members benefited from formal and informal peer support and reassurance when dealing with challenging cases and a busy workload. The sense of contributing to the important national public health effort to keep schools open galvanized team members during challenging situations and promoted a positive work ethic. This was reflected in individuals' willingness to adapt their work practices significantly (e.g., longer days, weekends) compared to their previous posts.

This survey also identified factors which acted as barriers to effective multisectoral working. Conflicting media messaging and rumors of potential changes to COVID-19 guidance caused frustration among Schools Team members when communicating with school principals. Standardized protocols were updated regularly to reflect changes in national guidance. However, the frequency of these updates was identified as a source of frustration among principals and required regular re-training sessions for Schools Team staff. The formation of the Schools Team occurred during waves two and three of the COVID-19 pandemic in Ireland, and the associated additional workload for the Public Health department was a challenge. Consequently, the level of administrative support available to the Schools Team was reduced at times resulting in late or duplicate COVID-19 case notifications, which caused frustration among team members and confusion among principals. Despite these challenges, the majority of team members stated that they were willing to work with the Schools Team again in the future, reflecting the successful integration of various disciplines into a single effective team.

### 4.2. Strengths and limitations

The high response rate (53/70, 75.7%) and number of respondents to the online questionnaires (*n* = 53) add to the validity of the findings and were above average for similar mixed methods investigations (21, 22).

However, in common with similar survey based evaluations, this dataset was susceptible to a number of sources of potential bias. No information was collected about the characteristics of non-responders and it was not known if this group differed systematically from those who did respond. However, the high response rate may have mitigated the risk of responder bias.

Semi-structured interviews were conducted over video call and were thus more susceptible to social desirability bias compared to written questionnaires, despite assurances given to interviewees that no identifiable information would be retained. The small number of semi-structured interviews (*n* = 4) limits the generalizability of the insights gained from this process.

While this evaluation was conducted specifically on the Schools Team in the East of Ireland, the formation of Schools Teams was a national public health initiative, with similar teams established in the other Public Health departments in Ireland. It is, therefore, possible that the barriers and facilitators to effective multisectoral team working identified by this study may not be generalizable to the experiences of Schools Teams working in other regional Public Health departments.

The assessment of the effectiveness of the Schools Team to mitigate SARS-CoV-2 transmission was analyzed in terms of the subjective experiences of schools team members only. No quantitative data to this effect was collected in this study.

### 4.3. The schools team model as an enabler for health system resilience

The COVID-19 pandemic has highlighted the importance of ensuring that health systems can “prepare for, manage and learn” from severe shocks (23). The multisectoral Schools Team model provides a framework for how the expertise and capacity of allied sectors may be recognized and leveraged to increase health system resilience in the face of unforeseen shocks. The Schools Team exhibited several characteristics associated with health system resilience, as outlined below, which may be used when designing future similar initiatives (23, 24).

The flexibility demonstrated by all stakeholders during the initial establishment of the Schools Team, at both national and regional level, ensured the most efficient use of resources available to the health system. This whole of government approach allowed the Public Health and Education sectors to adapt to an acute shock without sacrificing the provision of core services. Effective governance allowed national co-ordination of roles and responsibilities between various stakeholders. Such leadership ensured consistent best practice operating protocols were implemented by all Schools Teams nationwide and avoided the fragmented approach associated with less resilient and inefficient organizations (25).

Effective communication between Schools Team personnel at the national and regional levels was promoted by regular reporting of COVID case numbers and timely dissemination of updates to operating protocols. This efficient information flow was a key facilitator of informed decision making among Schools Team leadership and ensured that the overall team objectives were communicated across all stakeholders. The resulting culture of open communication encouraged staff to share new ideas to improve the effectiveness and relevance of team protocols and is recognized as a vital factor in organizational resilience (26, 27).

This study also demonstrated that a congenial work culture not only facilitated effective collaboration, but meant that Education sector staff were willing to work with Public Health, if required, in the future. This indicates the strength of the working relationships formed between Education and Public Health during the pandemic response. These relationships need to be maintained and nurtured in the post-pandemic recovery phase to ensure that the connections are not lost and that future collaboration between the sectors will be possible to address new health threats that may emerge. Similarly, as public health practitioners endeavor to address the challenges posed by complex population health problems in the post-pandemic phase, such as the climate crisis, obesity, and the growing burden of mental health disorders worldwide, a holistic approach beyond Public Health alone will be required.

### 4.4. Application of the Schools Team model to other public health priorities

Despite the advantages of multisectoral partnerships and their potential to benefit population health, this model remains uncommon within public health practice. This deficit may be due to either a lack of evidence to demonstrate the benefits of collaborative working, lack of mechanisms to support similar initiatives, lack of multisectoral accountability for health issues or lack of awareness among public health practitioners and senior health managers. An absence of similar teams in other international jurisdictions precluded comparison of the Schools Team model against existing benchmarks and highlighted the need to improve the evidence base of multisectoral and interdisciplinary working by ensuring service evaluations are integrated into future initiatives. The potential for the application of the multisectoral Schools Team model to other complex public health priorities are considerable.

The strong links established between the public health and education sectors to develop the Schools Team model should be extended and strengthened to more effectively pursue specific public health priorities in school settings. Two such priorities we suggest are school-based vaccination uptake and health promotion initiatives. Pandemic related disruption of school based vaccination programmes have contributed to the ongoing public health threats posed by vaccine preventable diseases, e.g. measles, polio. Lessons learned from the Schools Team initiative are particularly relevant in the application of multisectoral partnerships to optimize vaccine uptake in schools, and inform further collaborative working between public health and education sector professionals to ensure consistent vaccine-related communication and delivery in school settings. Such initiatives would also provide a vital resource to address vaccine mis-information which may be targeted at parents and schools.

The lessons highlighted by this study also extend to non-infectious disease public health priorities in school settings. The creation of environments supportive of health and the development of personal skills are cornerstone components of the Ottawa Charter for Health Promotion and schools represent a key setting where these components can be delivered (28). Effective collaboration between Education and Public Health sector professionals should support development and enhancement of health promotion initiatives to make the healthy choice (e.g., diet, exercise, active transport) the easy choice for students and staff alike.

The Schools Team model could also guide the formation of linkages between Public Health and non-Education sectors to proactively address public health priorities outside of the school setting. Given the growing complexity of modern infectious disease threats, initiatives could include the development of a formal collaborations with government and non-governmental organizations, to enhance efforts to mitigate disease transmission in congregate settings such as accommodation centers for refugees. The ongoing war in Ukraine and resulting mass population displacement has highlighted the need for a such a coordinated approach between Public Health and other relevant sectors in this regard (e.g., justice and social protection departments). Collaboration with agricultural sector colleagues could also be promoted to support the One Health approach in balancing the health of people, animals and the environment, and protect the population against increasingly complex health threats across these domains.

## 5. Conclusion

The increasing scale of public health concerns underscores the need to better understand and promote factors which contribute to health system resilience. The Schools Team model illustrates the potential of multisectoral partnerships to effectively address complex public health priorities. However, increased awareness of this model is needed among public health practitioners and policy makers if this potential is to be realized.

The factors which contributed to the success of this initiative provide vital learning to enhance the ability of health systems to maintain core services in the face of unforeseen acute shocks. More broadly, mechanisms to support multisectoral working should be developed, monitored and evaluated expanding beyond reactive interventions to proactively address key health priorities which foster recovery and build resilience across health systems and communities. Such collaborations would promote healthier populations by promoting and encouraging a public health perspective among other sectors and embedding “health in all policies”.

## Data availability statement

The raw data supporting the conclusions of this article will be made available by the authors, without undue reservation.

## Ethics statement

Ethical review and approval was not required for the study on human participants in accordance with the local legislation and institutional requirements. Written informed consent for participation was not required for this study in accordance with the national legislation and the institutional requirements.

## Author contributions

PN, CK, PW, and EK drafted the original manuscript. PN, EK, and AH contributed to acquisition of data. Data analysis and visualization was carried out by PN. All authors contributed revisions to the manuscript and approved the final manuscript for publication.

## Footnotes

^1^ https://www.who.int/news/item/29-06-2020-covidtimeline

^2^ https://data.unicef.org/resources/one-year-of-covid-19-and-school-closures/

^3^ https://www.ecdc.europa.eu/en/publications-data/children-and-school-settings-covid-19-transmission#no-link

^4^ https://web.archive.org/web/20200919034854/https:/www.gov.ie/en/publication/2dc71-level-5/

^5^ https://www.jotform.com/

^6^ https://www.qsrinternational.com/nvivo-qualitative-data-analysis-software/home

^7^ https://www.irishstatutebook.ie/eli/1981/si/390/made/en/print
