# Supplementary material for: Lessons from inter-disciplinary collaboration to mitigate SARS-CoV-2 transmission in schools, Ireland, 2020/2021, to inform health systems and multisectoral recovery
Source: Front Public Health. 2023 Jan 16;10:1072566. doi: 10.3389/fpubh.2022.1072566 (PMC9885185; doi:10.3389/fpubh.2022.1072566)
Supplement: Supplementary file 1 [file Data_Sheet_1.PDF]

Schools Team Feedback Questionnaire

Dear Participant

The Department of Public Health (HSE-East) is currently undertaking a review of our response to COVID-19 in educational settings for the 2020/2021 academic year. As part of this review, we are asking staff members to share their experiences of working with the Schools Team during this time. All responses are completely anonymous and no identifiable information is collected. This survey will take approximately five minutes to complete. Thank you for your time.

1. Please indicate your role within the Regional Schools Team

- ◯ Administration
- ◯ Department of Education staff member
- ◯ Doctor
- ◯ Nurse
- ◯ Allied Health
- ◯ Other

2. Where was the majority of your work delivered

- ◯ Onsite (HSE site)
- ◯ Onsite (Department of Education site)
- ◯ Remotely
- ◯ Mixture

3. How long did you work with the Regional Schools Team

- Less than 1 month
- Between 1 and 2 months
- Between 2 and 5 months
- Longer than 5 months

4a. Internal Communication (communication within the Regional Schools Team)

	Strongly agree	Agree	Neutral	Disagree	Strongly disagree	Not applicable
Communication within the Regional Schools Team was clear (e.g. roles, responsibilities, instructions)	○	○	○	○	○	○
Methods of communication used within the Schools Team were efficient (e.g. huddles, team meetings, e-mail updates)	○	○	○	○	○	○
I was able to contact my supervisor/Lead/Line manager in a timely manner as required	○	○	○	○	○	○

4b. What were the strengths of the internal communication process within the Regional Schools Team?

.

4c. List any suggestions to improve internal communications within the Regional Schools Team.

Type here...

5a. External Communication (communication from Regional Schools Team to other stakeholder)

	Strongly agree	Agree	Neutral	Disagree	Strongly disagree	Not applicable
Communication from National to Regional Schools Team was clear	○	○	○	○	○	○
Communication from Regional Schools Team to principals or managers was clear	○	○	○	○	○	○

5b. What were the strengths of the communication between the Regional Schools Team and external stakeholders (e.g. principals)?

Type here...

5c. List any suggestions to improve communications between the Regional Schools Team and external stakeholders.

Type here...

6a. Standard Operating Procedures and Training

	Strongly agree	Agree	Neutral	Disagree	Strongly disagree	Not applicable
I received formal training before commencing my role	○	○	○	○	○	○
My role was well defined within the Schools Team	○	○	○	○	○	○
I was provided with written standard operating procedures	○	○	○	○	○	○
Updates to standard operating procedures were communicated in a timely manner	○	○	○	○	○	○
I had adequate resources to complete the tasks assigned to me	○	○	○	○	○	○
I was comfortable with my level of responsibility within the Schools Team, following training	○	○	○	○	○	○

6b. What were the strengths of the standard operating procedures within the Schools Team?

Type here...

6c. List any suggestions to improve the standard operating procedures within the Schools Team

Type here...

7. General

	Strongly agree	Agree	Neutral	Disagree	Strongly disagree	Not applicable
There was a culture of support, openness and respect within the Schools Team	○	○	○	○	○	○
I would be happy to work with the Schools Team in the future	○	○	○	○	○	○
I felt the Schools Team was able to effectively support schools to reduce transmission of COVID-19	○	○	○	○	○	○

8. Are there any other comments you would like to add about your experience working with the Schools Team?

Type here...

Submit

Now create your own Jotform - It's free!

Create your own Jotform
